# Graph-Based Analysis for the Characterization of Corrugated Board Compression

**DOI:** 10.3390/ma17246083

**Published:** 2024-12-12

**Authors:** Taieb Belfekih, Ricardo Fitas, Heinz-Joachim Schaffrath, Samuel Schabel

**Affiliations:** Chair of Paper Technology and Mechanical Process Engineering, Technical University of Darmstadt, 64289 Darmstadt, Germany; taieb.belfekih@stud.tu-darmstadt.de (T.B.); heinz-joachim.schaffrath@tu-darmstadt.de (H.-J.S.); samuel.schabel@tu-darmstadt.de (S.S.)

**Keywords:** graph-based structural analysis, dimensionality reduction, corrugated boards, image analysis, compression behavior

## Abstract

This paper proposes a novel approach to represent the geometry of the corrugated board profile during compression using graphs. Graphs are lighter than images, and the computational time of compression analysis is then significantly reduced compared to using the original image data for the same analysis. The main goal of using such graphs is to gain more knowledge about the mechanical behavior of corrugated boards under compression compared to the current load–deformation curve approach. A node tracking algorithm is applied to characterize the different phases occurring during the compression test in order to predict physical phenomena, including buckling and contact. The main results show that analyzing the nodes provides significant insights into the compression phases, which has not been achieved in the current state of the art. The authors believe that the objective of this research is crucial to better understanding the physics of corrugated boards under compression, and it can also be extended to other engineering structures.

## 1. Introduction

Corrugated board is one of the most popular and in-demand materials in the packaging sector. It is lightweight and affordable, and offers good protection and cushioning for objects being transported [1,2]. Additionally, it provides effective and sustainable packaging options that are in great demand across a variety of industries, including e-commerce, pharmaceuticals, cosmetics, food, and beverage [1,3,4]. It is a highly recyclable and biodegradable material as well. Enhancing the understanding of corrugated board mechanical behavior under compression aids in product design optimization by lowering the product’s weight and cost and by boosting its capacity to absorb energy. Packages with such ideal designs are less expensive and lighter yet are able to provide cushioning. Another area of optimization that can be studied is the prediction of energy absorption and corrugated board failure.

Shape and topology optimization in corrugated boards has not yet been thoroughly examined in the literature [1], and modeling approaches to tackle those challenges should be investigated since these may enhance the robustness and applicability of the findings. In the optimization process, reliability in terms of computational efficiency is crucial. Finite element models were utilized in earlier studies [5,6] to simulate deformation and analyze stress in the corrugated board under out-of-plane compression. The Flat Crush Test (FCT) is the equivalent test. While being accurate, the finite element method is computationally expensive. Also, the only reliance on FEM modeling that dominates in the field of corrugated board would make the accuracy and reliability of optimization solutions strongly dependent on the precision of models [1], where the fluting medium is approximated with an ideal sine wave, which, for example, does not replicate the symmetry imperfections in the real sample, due to the sole reliance on FEM modeling that is dominant in the field of corrugated boards. The experimental testing of samples yields a load–deformation curve that characterizes the general behavior of the board. It does not, however, provide additional information about the compressive behavior of the corrugated board, such as when or how buckling occurs due to contact between the liners and the flute.

An image analysis could be investigated in order to close the gaps described earlier. It is not ideal to use raw photos in optimization studies since they contain an excessive amount of data that need to be stored and analyzed. Consequently, it can be beneficial to reduce the dimensionality of picture data by summarizing the most crucial information in the image using graphs. A similar goal of dimensionality reduction is achieved in the literature using graphs [7,8] to effectively describe data distribution or structure. The efficient analysis of corrugated board images in terms of both time and data storage is carried out by taking into account the graph representation of the board geometry. Compared to raw photos, graphs contain a lot less data, but they provide more about the profile shape than the experimental load–deformation curve does. They serve to condense the profile geometry, which represents the most significant piece of information in the image. Additional information about the deformation and buckling behavior of the fluting structure can be obtained by tracking the nodes in the graphs and calculating their displacement. Similarly, other recent works attempt to use graph-based methods as a direct replacement for the FEM, as *“Computational time can be prohibitive”* [9]

In this work, a novel method for graph-based image analysis of corrugated boards under compression is proposed. An image of the profile geometry reproduces the symmetry defects of the fluting medium based on pictures of actual samples under compression.

The aims of this method are as follows:

- Reducing the dimensionality of corrugated board images using graphs.
- Gaining more information about the mechanical behavior of corrugated boards in comparison to the load–deformation curve.

The remainder of this paper is as follows: In Section 2, the state of the art about image analysis in corrugated boards and in other fields is presented; Section 3 presents the materials and methods that are used in this paper; Section 4 presents and discusses the results achieved based on the applied methodology and the limitations tackled; and Section 5 summarizes the most relevant of the results and makes reference to future works.

## 2. Related Works

The selected papers presented in this section are based on a selection of keywords and querying tasks in major scientific databases such as Web of Science, Scopus, and Google Scholar. For the first subsection, the keywords were “Corrugated” AND (board OR fiberboard OR cardboard) AND (“Image analysis” OR “Image processing” OR “Graph” OR “Graphs” OR “Image segmentation” OR “Cross-section images” OR “Profile images” OR “Machine vision” OR “Computer vision”). For the second subsection, in order to include other structural problems, keywords related to corrugated boards were replaced with (“structural analysis” OR “mechanical analysis” OR “mechanical behavior”). For the third subsection, where graphs-based solutions are emphasized, the following were used: (“structural analysis” OR “mechanical analysis” OR “mechanical behavior”) AND (“Graph” OR “Graphs”). Inclusion criteria also involved recent research papers, especially in general structural problems not related to corrugated boards, that were published from 2023. Exclusion criteria included papers where mechanical or structural engineering problems were not considered.

### 2.1. Works Concerning Corrugated Board Structural Analysis

An image analysis based on the genetic algorithm was applied to corrugated board profiles in a recent work conducted by Rogalka et al. [10]. The goal was to calculate an estimation of geometric features, namely the thicknesses of different layers, centerline, period, and initial phase of the fluting medium that was approximated with a sine wave. Later, the same analysis was applied to double-walled corrugated board [11]. Geometric features could be identified precisely in the case of non-crushed board but with less precision when the same procedure was applied to crushed samples. The effectiveness of the procedure depends heavily on the quality of the sample cross-section. The same authors applied a Convolutional Neural Network (CNN) for the classification of seven different types of corrugated board [11]. The algorithm was highly efficient and achieved, in some cases, more than 99% accuracy. The classification of crushed samples was also possible, highlighting the robustness of CNN applications. In a later work, two classification methods of corrugated boards based on cross-section images were compared. The first method involved using a genetic algorithm to identify geometric features alongside a simple feedforward neural network. The second method was based on the already mentioned CNN application. The second method was found to be more efficient in both precision and inference time [12].

Still related to corrugated boards, refs. [13,14,15] developed algorithms for the automatic counting of stacked corrugated boards based on images of the profile side of corrugated board stacks and also the slitter side, where the corrugation is not depicted. The results of the developed procedures are satisfactory and can potentially assist in automating the counting task for the production and quality control of corrugated boards. Later, an algorithm was developed to combine corrugated board images using image stitching techniques and feature tracking. This method was applied to images of the profile side of corrugated board stacks. For corner detection, the Forstner method was used. The RANSAC method was applied to correspond to the features detected. Images were combined using bundle adjustment. The results showed that cardboard images were correctly combined when at least 25% overlap was present. In other works, the number of board sheets was also correctly calculated based on the stitched image [14].

### 2.2. General Structural Analysis Problems Involving Image Analysis

In other words, not related to corrugated boards but still related to the method presented here, methods of image analysis were applied for the deformation characterization of materials under given load or displacement conditions. An algorithm based on Digital Image Correlation (DIC) was proposed by Radi et al. [16] for the deformation tracking of truss lattices under dynamic loading. Depending on the truss type, a grid was generated to define points of interest in the structure. During a compression test of the structure, the nodal displacement of these points of interest was tracked using a DIC-based algorithm. It was shown that optimal results highly depend on the choice of imaging frame rate. Measurement error was shown to be under 1 pixel for the tested trusses, highlighting the efficiency of the procedure.

Image analysis methods were also used to track the deformation in honeycomb structures. As an example, the work of Hu et al. [17] is mentioned. Image skeletonization and branch point matching algorithm were used to track the deformation of honeycomb structures under compression and tension. Calculating the mean bias errors in the simulations showed that the algorithm attains sub-pixel precision. Additionally, the correct matching of branch points on branches with imperfections was achieved, proving the accuracy and robustness of this procedure when applied to honeycomb structures.

Liang et al. [18] developed an image analysis procedure to determine the curvature of continuous fiber-reinforced material in a bending test. It was based on the binarization and skeletonization of the transverse cross-section image of a sample during a bending test. The curved geometry of the material was represented by approximating some points of interest on the cross-section using a uniform quartic B-spline. After the curvature values are determined, a bending moment–curvature curve was successfully generated.

Mixed FEM and computer vision approaches were also utilized by Xie et al. [19]. Here, a seismic evaluation of a 30-meter-tall sculpture constructed of Western Red Cedar logs was conducted to illustrate a novel approach that combines computer vision and 3D point cloud methodologies for the accurate structural analysis of irregular timber structures. The proposed method uses consumer-grade devices to capture images, which are subsequently converted into three-dimensional (3D) models using photogrammetry software. In taking into consideration uneven geometries and variable stiffness, the revised finite element models obtained from these scans provide noticeably higher accuracy than standard equivalent models. However, the limitations pointed out are related to a lack of trained personnel for the creation of FE models, as well as the implementation of advanced point cloud registration algorithms for handling automatically multiple scans.

Another study conducted by Dutta et al. [20] looked at the effects of manufacturing flaws such as gaps and overlaps on the thermal buckling behavior of variable-angle tow-steered composite laminates. The study found and examined prepreg tape layer flaws and assessed their effect on structural performance using a novel combination of image processing and reduced-order modeling. A Proper Orthogonal Decomposition approach was used to reduce computational complexity in a dense finite element model that is based on binary matrix representations of images. The findings showed that, in comparison to quasi-isotropic laminates, VAT laminates with non-linear fiber pathways can increase the critical buckling temperature by up to 37.56%. Overlaps highlight a trade-off in defect control because they increase plate weight while also improving stiffness and buckling resistance. The impact of reduced-order modeling for structural analysis is clearly evident from this work.

Chen et al. [21] presented a computer vision-based approach for transmission tower damage identification and structural analysis, with a particular emphasis on the bending detection of tower members through processes like curve fitting, edge and contour detection, and mask creation. Using image-based evaluations, the method showed strong performance in recognizing damaged parts and their bending states. Furthermore, by developing a coefficient that measures the correlation between damaged and impacted components, the study incorporated finite element analysis to assess the effect of damaged elements on the tower’s overall structure. The findings showed that members below cross arms, especially in the center of the tower, were more vulnerable to related damage and needed to be inspected and maintained more frequently. Significant advancements in structural analysis and transmission tower maintenance techniques are possible with this technology, which offers an economical and effective tool for field inspections. This was made possible by incorporating computer vision into the structural analysis problem-solving process rather than depending exclusively on FEM-based techniques.

In the field of nanostructures, Chen et al. [22] examined the degradation of GaN-based LEDs subjected to saline mist conditions. The study monitored and reduced LED deterioration by combining innovative circuit design, artificial intelligence, and machine vision. When a failure was discovered, a new system that used YOLO V8 for real-time damage monitoring and automated circuit switching guaranteed continuous operation by switching to backup LEDs. The effect of corrosion caused by NaCl on LED performance was revealed through thorough material characterization, which included SEM, TEM, and XRD investigations.

Luo et al. [23] suggested a unique way for combining image processing and a multi-scale FEM to forecast the mechanical behavior of composite materials, particularly concrete. The technique segments aggregates, mortar, and air voids using CT scan pictures of laboratory-prepared concrete samples that have been processed with MATLAB. In ABAQUS, these segmented images were transformed into finite element models, with distinct attributes defined for every phase. The FEM’s correctness was confirmed by experimental compression tests, which revealed an inaccuracy of just 4% when compared to experimental findings. This method offers excellent accuracy and efficiency for simulating heterogeneous composite materials while drastically cutting down on the time and expenses related to conventional empirical testing.

A study conducted by Jung et al. [24] presented a deep learning-based super-resolution technique using a residual neural network (SRResNet) to enhance the resolution of low-resolution (LR) digital microstructure images, such as those obtained from EBSD (Electron Backscatter Diffraction) data. The method reconstructs high-resolution (HR) images from LR inputs with accuracy comparable to experimental HR data, enabling detailed microstructure characterization and finite element mechanical analysis. This approach overcomes limitations of conventional interpolation methods, preserving critical features like grain boundaries and phase interfaces, which is essential for studying material behavior such as stress localization and mechanical performance. The SRResNet demonstrated superior performance in both synthetic and experimentally acquired datasets, offering a rapid, accurate, and resource-efficient alternative for material science applications, showing that it is possible to maintain accuracy using more efficient, image-based methods.

### 2.3. Graph-Based Solutions in General Structural Analysis Problems

Using the aforementioned methodology, the authors found two very recent works where graph theory was applied for solving structural analysis problems.

Block et al. [25] investigated how to improve the evaluation of structural redundancy in frames by combining structural mechanics and graph theory. Beyond conventional metrics like stresses and strains, the authors addressed the geographical distribution of static indeterminacy by integrating the Maxwell–Calladine counting rule, the redundancy matrix, and graph theoretical techniques. Through quantifying the load-independent redundancy distribution, the redundancy matrix provides information about the performance and structural integrity of the system. Graph theory supports this using bipartite graphs to visually represent connectedness and redundancy. The potential for strong and effective structural design in the early phases of planning is highlighted by the demonstration of this interdisciplinary method on trusses and grid structures.

Kiær and Liabø [26] investigated how to combine Graph Neural Networks (GNNs) with FEM in a parametric design framework for structural analysis. Using GNNs to improve computing efficiency during structural behavior predictions, the authors created the Velociraptor framework, which consists of machine learning models (ArchNN and TrussNN) and FEM tools (Velociraptor2D and Velociraptor3D). In addition to FEM’s accurate benchmarking, the framework provides quick, real-time computations during design processes using GNNs for tasks like forecasting nodal moments in arches and time histories for truss structures. This method shows how integrating FEM and AI can improve workflows and increase AI’s usefulness in structural engineering.

The fact that there are few works published in this field underscores the importance of developing new methodologies involving graph theory to solve structural problems in the future.

## 3. Materials and Methods

### 3.1. Graphs

Graphs are a type of data that help define and visualize relationships between various components. They are used in various fields like mathematics, computer science, and data science. A graph is a set of nodes and edges between some node pairs. As defined by Diestel [27],

“A graph is a pair G=(V,E) of sets such as $E\subseteq {\left[V\right]}^{2}$; thus, the elements of *E* are 2-element subsets of *V*. The elements of *V* are vertices (or nodes, or points) of the graph *G*, the elements of *E* are its edges (or lines).”

Some of the main terminology in graphs are the concepts of order, neighbors, degrees, and paths. The order of a graph is equal to the total number of nodes it contains, i.e., the number of elements in *V*, and it is denoted by |G|. The number of edges is denoted by ∥G∥ [27].

Two vertices *x* and *y* are neighbors if (x,y) is an edge in *E* [27]. The degree of a vertex *v* is written as $d_{G}\left(v\right)$ and is equal to the number of edges related to *v*. By the already mentioned definition of a graph, the degree of a node is always equal to the number of neighbors it has [27]. This would not be valid in the case of multigraphs, where more than one edge is allowed between two nodes.

A path defined as a non-empty graph of the form $V=\{x_{0},x_{1},\ldots ,x_{k}\}$ and $E=\{x_{0}x_{1},x_{1}x_{2},\ldots ,x_{k-1}x_{k}\}$, where $x_{0}$ and $x_{k}$ are the ends of the path. In more common sense, a path is the sequence of nodes that lead from a node $x_{0}$ to a node $x_{k}$. The length of a path is equal to the number of edges that are traversed when the path is followed [27].

Some of the principal types of graphs are directed vs. undirected, connected vs. disconnected, weighted, and simple graphs. A graph is connected if there exists at least one path relating to every two nodes in that graph. Otherwise, the graph is disconnected ([28], p. 6). Directed graphs have edges with a specific direction so that the edges (u,v) and (v,u) are not equivalent. In undirected graphs, edges do not have a specific direction. As a result, (u,v) and (v,u) are equivalent. Weighted means that different numbers are attributed to the edges. In unweighted graphs, all edges are considered equally weighted by 1. A graph is simple if there are no self-loops, i.e., edges of the form (u,u), or multi-edges, i.e., more than one edge between two nodes [29].

**Figure 1:**
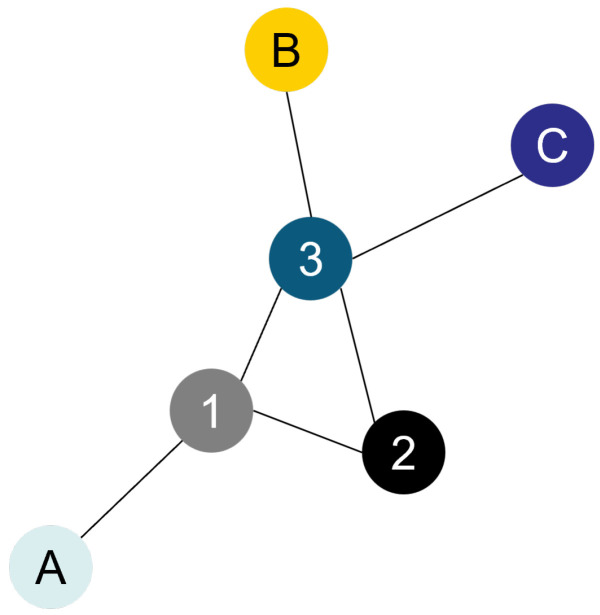
Example of an undirected, connected, and simple graph.

An example of an undirected, connected, and simple graph is illustrated in Figure 1. Its order is 6, which is the number of nodes it has. The sets of vertices and edges are, respectively, as follows:

V={1,2,3,“A”,“B”,“C”}

E={(1,“A”),(1,2),(1,3),(2,3),(3,“C”),(3,“B”)}

For example, the set of neighboring nodes of node 1 is {“A”,2,3}, and the degree of node 1 is then $d_{G}\left(1\right)=3$.

### 3.2. Methodology

**Figure 2:**
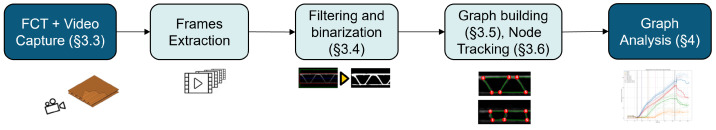
Flowchart representing the methodology followed in the current study.

A methodology to reduce the data dimensionality of corrugated board images by employing graphs is proposed and presented in this section. The flowchart in Figure 2 illustrates the steps of the methodology. In Section 3.3, the gathering method of the experimental data is presented. A video was acquired using a camera, and images were extracted from the video and subjected to pre-processing and filtering. The filtering process is presented in Section 3.4. In Section 3.5, the process of obtaining the graphs from filtered images is detailed. Section 3.6 details the graph filtering and node tracking methods. Section 3.7 discusses the choosing of parameters of the image filtering and binarization processes when visualizing the resulting graph.

### 3.3. Experiment Setup

**Figure 3:**
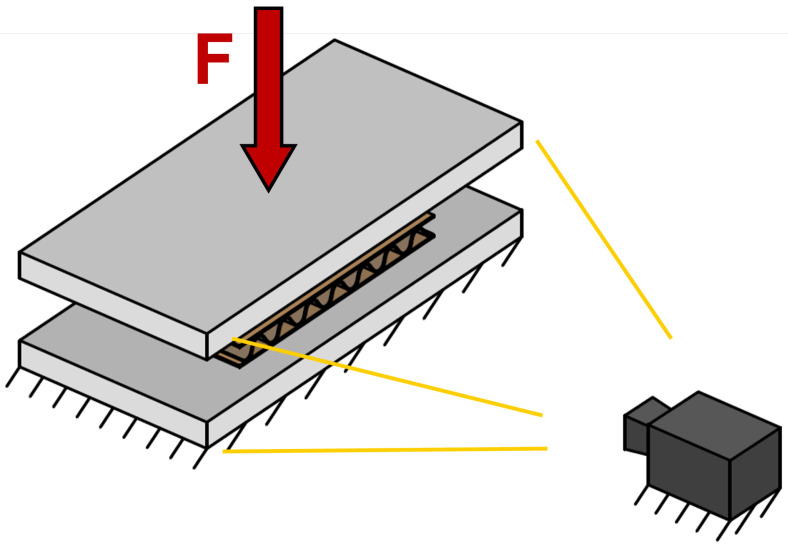
Simple representation of the experimental setup.

A compression test was performed on corrugated board samples, which were loaded perpendicular to their surface. A Zwick machine (ZwickRoell GmbH & Co. KG, Ulm, Germany) was used for this purpose. A grayscale camera was mounted on a frame that was connected to the machine housing. The model of the camera was “Vosskühler HCC—1000/512 s” (Osnabrück, Germany). An objective lens “NIKKOR 50 mm 1:1.4” with a focal length of 50 mm and a Teleconverter “Nikon TC—201 2×” (Tokyo, Japan) to double the focal length were used. A video of the corrugated board cross-section during the compression test was recorded. It had a resolution of 256 × 1024 pixels. One pixel represents 87 ± 2 μm. A self-made LED light source was used to illuminate the profile of the specimen. It was made of 2 sets of LEDs, each with a maximum power of 100 W. A laboratory generator was used as a power supply for the LED lights. The voltage was set to 28 V, which resulted in a power of 40 W. Camera and lens parameters, like, for example, the Depth of Field (DoF) and the exposure time, were manually adapted to obtain a sharp and well-illuminated image. A simplistic representation of the process is shown in Figure 3.

**Figure 4:**
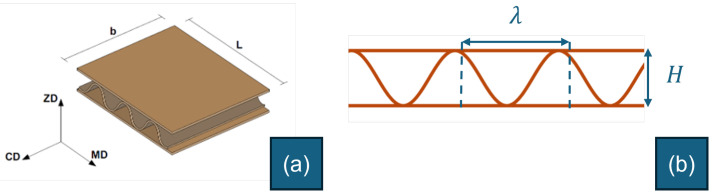
Dimensions of the corrugated board sample: (**a**) 3D view; (**b**) 2D profile.

Samples were conditioned in laboratory conditions of humidity and temperature of 50 ± 2% and 23 ± 1 °C, respectively. Tests were performed in the same laboratory conditions and under LED lights only. This way, the videos recorded have a dark background, and the thin-walled structure of the corrugated board profile is well depicted. The compression test was performed using a prescribed displacement, with a constant movement speed of the upper compression plate set at 5 mm/min. Corrugated board samples were cut, using a saw machine, into a rectangular shape with specific dimensions, as shown in Figure 4. The length of the samples was *L* = 100 mm, consisting of several waves of the corrugated medium, and the width was *b* = 25 mm. The shape of the samples was chosen to be rectangular rather than circular so that the board profile, which was the object to be captured, was a flat surface with a constant normal distance from the camera.

**Figure 5:**
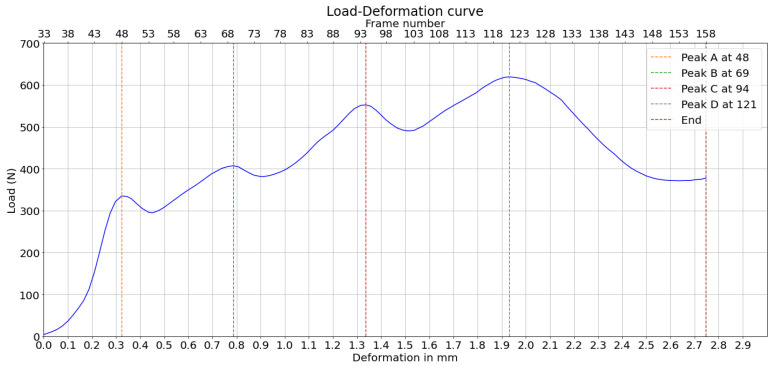
Load–deformation curve corresponding to the compression test being analyzed—Experiment 1.

**Figure 6:**
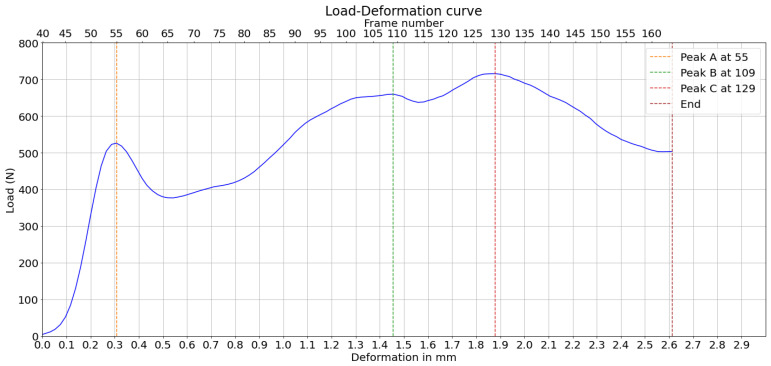
Load–deformation curve corresponding to the compression test being analyzed—Experiment 2.

Two corrugated board specimens were selected based on the quality of the cross-section, which showed less delaminated paper fibers, and the clarity of the compression video. These two examples demonstrate the feasibility of graph-based image analysis in corrugated boards under compression. Their load–deformation curves are represented in Figure 5 and Figure 6. The load peaks are marked for later analysis. Geometric dimensions and characteristics are summarized in Table 1, where *H* and λ are the height and wavelength of the board, respectively, as illustrated in Figure 4, $t_{f}$ is the thickness of the flute paper, and $t_{l}$ is the thickness of the liner paper.

### 3.4. Filtering Process

**Figure 7:**
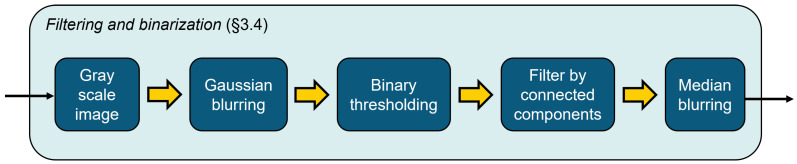
Filtering and binarization processes.

The main goal of the filtering process illustrated in Figure 7 is to eliminate paper fibers mostly coming from the damages of paper sheets when cutting the samples and appearing on the corrugated board cross-section image. Furthermore, the process aims at smoothing the board geometry and converting the image to binary, without altering the structure of the board. The process consists of Gaussian blurring followed by binarization based on a constant threshold. Subsequently, a connected component filtering is applied to remove isolated white regions with an area smaller than a given value in pixels. Finally, median blurring is used to smooth the surface geometry of the corrugated board.

The filtering constants and kernel sizes are set manually and must be adapted to other samples individually to obtain optimal results. These manual adjustments of the filters depend on the thickness of the paper sheets constituting the different layers, the amount of delaminated paper fibers appearing on the cross-section image, and the given light conditions. The default filter parameters are the following:

- Gaussian filter: kernel size set to 5 ×5 and sigma to 1.
- Binarization threshold set to 25.
- Median filter: kernel size set to 3 × 3.
- Filter by connected components: minimum size of white regions set to 150 pixels.

A morphological closing filter is replaced with median blurring at the end, which not only fills the small black regions in a white surrounding but also smooths the surfaces and limits of the board. The histogram equalizer and adaptive OTSU method thresholding were investigated, but they did not help improve the image quality.

### 3.5. Skeletonization and Graph Building

**Figure 8:**
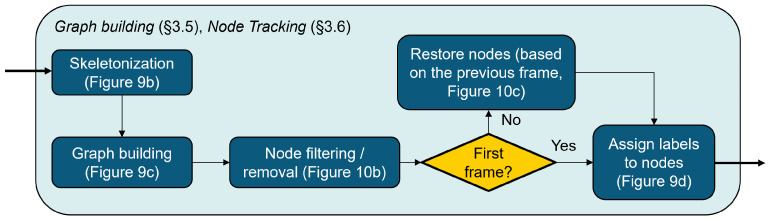
Graph building and node tracking processes.

The process that is constructed from the binary image to the filtered and labeled graph is summarized in Figure 8.

The skeletonization applied to the images of the corrugated board can be carried out with two different morphology functions from the Python library “Scikit-image”. The function names are “skeletonize” and “thin.” They use different algorithms to obtain skeletons of objects illustrated on binary images. Graphs are then built based on the obtained skeleton structure using the function “build_sknw” from the Python (3.11) library “sknw” [28] with the following inputs:

- **multi**: False, does not return a multigraph.
- **iso**: False, does not return a one-pixel node.
- **ring**: False, does not return self-loops, i.e., edges of the form (u,u).
- **full**: True, every edge starts from the nodes’ centroid.

**Figure 9:**
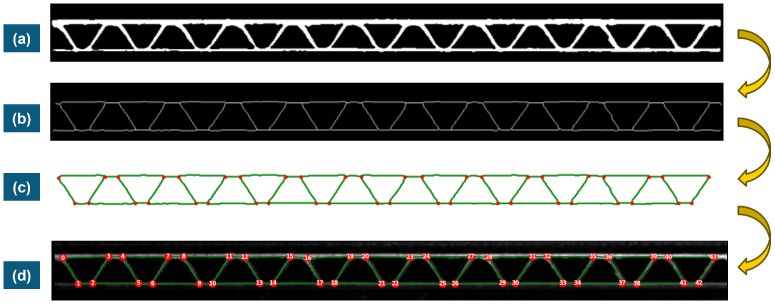
Process from image to graph. (**a**) binary image; (**b**) skeletonization; (**c**) graph with nodes and edges identified; (**d**) final graph with straight edges.

This function builds a graph, which is a Networkx object, based on a skeleton structure. “Networkx” is a Python library used to create and analyze graphs. Skeleton branches represent graph edges, and intersections between branches are the graph nodes. Intersections between branches in a skeleton structure are constituted of three pixels or more. One pixel, which is the centroid, is set as a node representing the intersection. When the parameter **full** is set to True, edges are related to the centroid of every intersection. Results of both skeletonization and graph building are also illustrated in Figure 9.

There are two ways to visualize the graphs obtained: using the skeleton branches as a visualization of edges or using straight lines relating between the nodes, as illustrated in Figure 9 (lowest two sub-images). Graphs are mostly visualized using nodes and straight-line edges.

### 3.6. Graph Filtering and Node Tracking

Simple graph filtering and node tracking are applied to every frame, and this process is now described. An iteration is carried out to delete all nodes with a degree equal to one, which means that they are related to only one other node. These nodes represent small paper fibers, which are not part of the structure. After removing these points, all nodes with a degree equal to two are also removed, unless they are endpoints of the graph at the left and right limits of the board. For node tracking, i.e., assigning labels to nodes based on previous frames, the graph obtained from the first frame is taken as a reference. To ensure reasonable results of segmentation and tracking, the reference graph created from the first frame should depict the geometry of the corrugated board profile correctly. In the case of the used specimen, the first graph created indeed builds a correct geometry of the profile. The nodes of the first graph are relabeled and numbered from the left to the right of the board to make the tracking visualization easier.

For node tracking, an iteration over all the nodes in the graphs of every two successive frames is carried out, where the distances between one node of the subsequent graph and all the nodes of the previous one are calculated. The minimum distance corresponds to the closest node from the previous graph. Nodes with a minimum distance are associated with each other, if not already associated.

**Figure 10:**
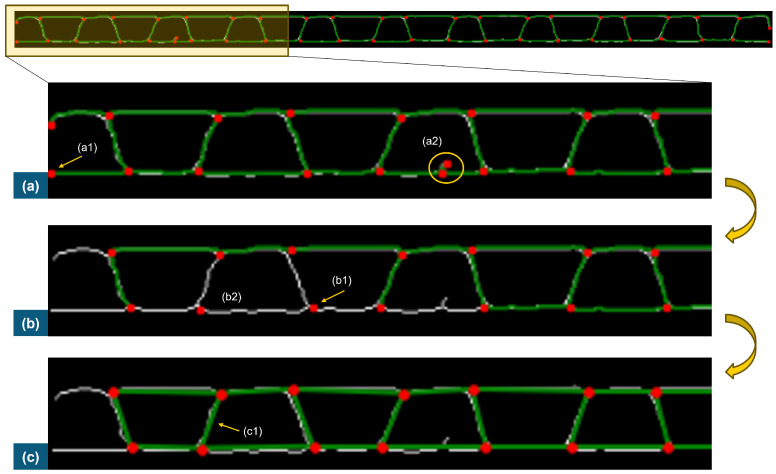
An example of how graph filtering is applied: (**a**) original graph, with extra nodes (**a1**) and (**a2**); (**b**) due to the node filtering process, edges are also removed (**b2**) and nodes from the previous frame are used instead (**b1**); (**c**) edge definition from the previous frame is used (**c1**).

During the compression test, the deformation happening can influence the images and, consequently, the results of filtering, skeletonization, and graph building. This can change the total number of nodes detected and the values of edges. In some frames, some nodes and edges cannot be detected, and in other frames, additional nodes and edges can emerge. When the number of nodes and the edge relations between them are not conserved, tracking problems can easily occur. Conserving the edge relations and the number of nodes along the frames of the compression video is necessary for the consistency of node tracking. A solution for this issue, which happens very often from frame to frame and especially when the nodes are very close to each other in the last frames corresponding to the end of the compression test, is suggested in the following four steps:

1. Nodes that are unique to the subsequent graph, i.e., additional nodes that emerge in the subsequent graph but were absent in the previous one, are eliminated because they are likely erroneous. This guarantees that the total number of nodes does not increase (Figure 10a).
2. Nodes unique to the previous graph and not appearing in the subsequent graph are added in the same position. This ensures that the total number of nodes does not decrease. (Figure 10b1).
3. All edges that are unique to the subsequent graph, i.e., edges that did not exist in the previous graph, are eliminated to prevent the formation of new relations between nodes, which would otherwise compromise the structure (Figure 10b2).
4. Edges unique to the previous graph, i.e., edges that correctly existed in the previous graph and are now absent in the subsequent graph, are added to ensure that the structure is not missing any segments (Figure 10c1).

**Figure 11:**
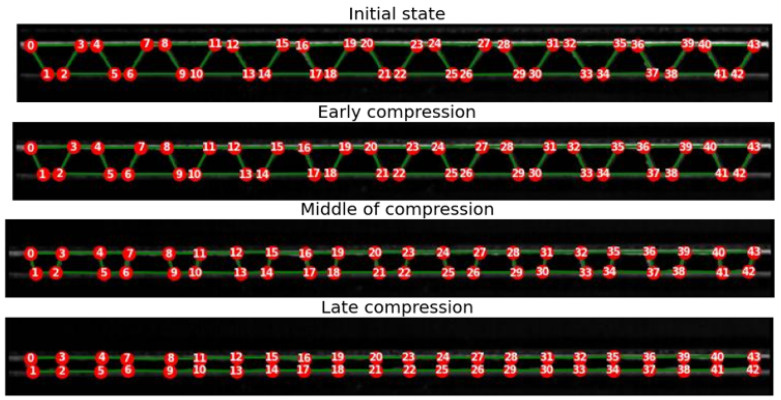
Results of the node tracking.

Results of the node tracking in some frames of the compression video are illustrated in Figure 11.

### 3.7. Parameterization of Image Filtering and Binarization

**Figure 12:**
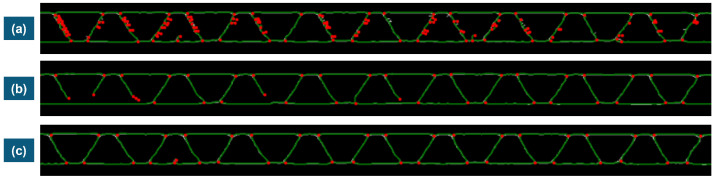
Resulting graphs of different selections of filtering parameters (**a**) Gaussian and Median Filter Kernels 1 × 1 and Binarization Threshold of 25; (**b**) Gaussian and Median Filter Kernels 5 × 5 and 3 × 3, respectively and Binarization Threshold of 50; (**c**) Gaussian and Median Filter Kernels 5 × 5 and 3 × 3, respectively and Binarization Threshold of 25. (Table 2).

The parameters listed in Section 3.4 were tested by trial and error until some satisfactory graphs were obtained. If these parameters are changed, one can observe some significant differences that will make it more challenging to proceed with the filtering or node tracking or, at least, make either the process less reliable or computationally more complex. In Figure 12, three different outputs are observed as an application of three different parameter settings listed in Table 2.

One can observe that (a) reducing the kernel size of both the Gaussian filter and median filter will result in several degree-one and degree-two nodes, which are not desired. Moreover, (b) increasing the binarization threshold will impact the real connectivity of the edges, which is also not desired. Therefore, (c) is a good compromise in that, even though it contains some nodes that need to be filtered, it becomes easier to remove them in the graph filtering part.

## 4. Results and Discussion

Even though more than two specimens were compressed, the authors now present the analysis of the insights given by two of those experiments. Each experiment corresponded to a specimen. In the future, more experiments from more specimen compressions will also be analyzed and published online. Please check the data availability statement for more information. Moreover, this section attempts to answer the research questions and discuss the limitations.

### 4.1. Image and Graph Analysis: Experiment 1

**Figure 13:**
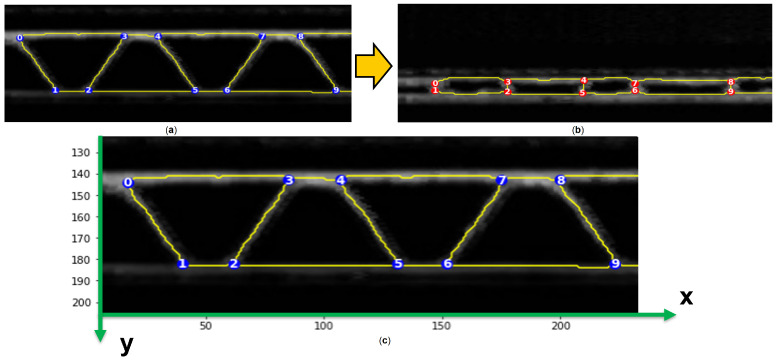
Visualization of the displacement vectors representing the displacement of the nodes between the initial (**a**) and final (**b**) frames. Displacement vectors are seen from nodes 1 to 11 (**c**).

In the specimen used for this experiment, 44 nodes numbered from 0 to 43 were detected and tracked over the frames of the compression video. The displacement computation of every node was calculated and referenced to its position in frame 33, where the compression starts. To visualize the displacement of nodes, displacement vectors were manually drawn in Figure 13 for nodes 0 to 11 between two frames extracted at the beginning and end of compression. The displacement computation of one node consists of calculating the magnitude of the displacement vector in every frame. It is also possible to calculate the magnitude of the horizontal component and the vertical component of the displacement vector separately, as illustrated for node 0. $d0_{x}$ represents the horizontal displacement of node zero, and $d0_{y}$ represents its vertical displacement between these two frames. This way, curves of the horizontal and vertical displacements can be obtained and observed separately. Based on this, both vertical and horizontal displacements of all 44 nodes are calculated over all the frames.

For any node *n* and any frame *f*, the absolute (or Euclidean) displacements $d^{\left(n,f\right)}$, horizontal displacements $d_{x}^{\left(n,f\right)}$, and vertical displacements $d_{y}^{\left(n,f\right)}$ are defined as follows:

$$\begin{array}{cc} d^{\left(n,f\right)} & =\sqrt{{\left(d_{x}^{\left(n,f\right)}\right)}^{2}+{\left(d_{y}^{\left(n,f\right)}\right)}^{2}},\\ d_{x}^{\left(n,f\right)} & =x^{\left(n,f\right)}-x^{\left(n,0\right)},\\ d_{y}^{\left(n,f\right)} & =y^{\left(n,f\right)}-y^{\left(n,0\right)}. \end{array}$$

**Figure 14:**
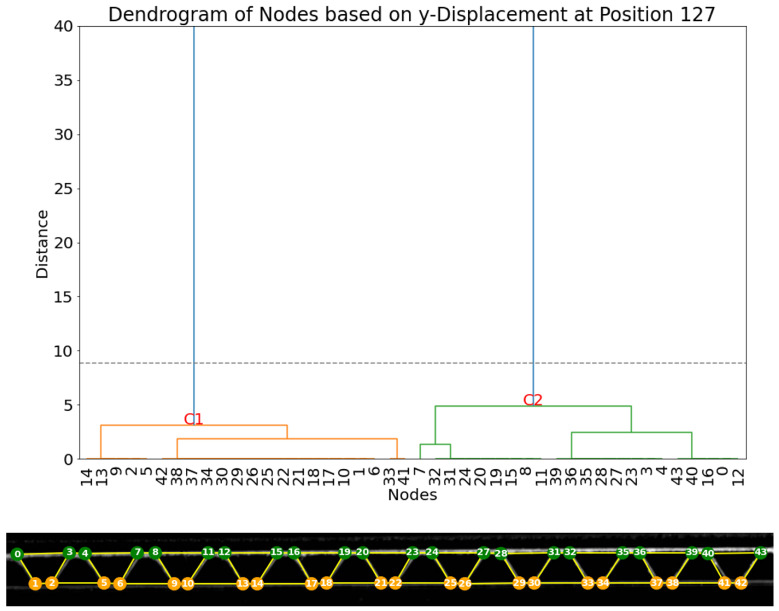
(**Top**): dendrogram and resulting clustering of the nodes based on vertical displacement $d_{y}$ in frame 127. (**Bottom**): visualization of the clusters (cluster 1: orange; cluster 2: green).

Firstly, the vertical displacements are analyzed. The dendrogram of displacements at frame 127 (already at the end of the compression), as shown in Figure 14, provides evidence for the existence of two principal clusters of nodes (C1 and C2). An examination of the original dataset of node displacements and visualization of node groups and subgroups in the same figure reveals that the two main clusters align with the two expected groups of nodes perfectly, as cluster C1 contains all 22 nodes within the lower liner and all 22 nodes within the upper liner.

**Figure 15:**
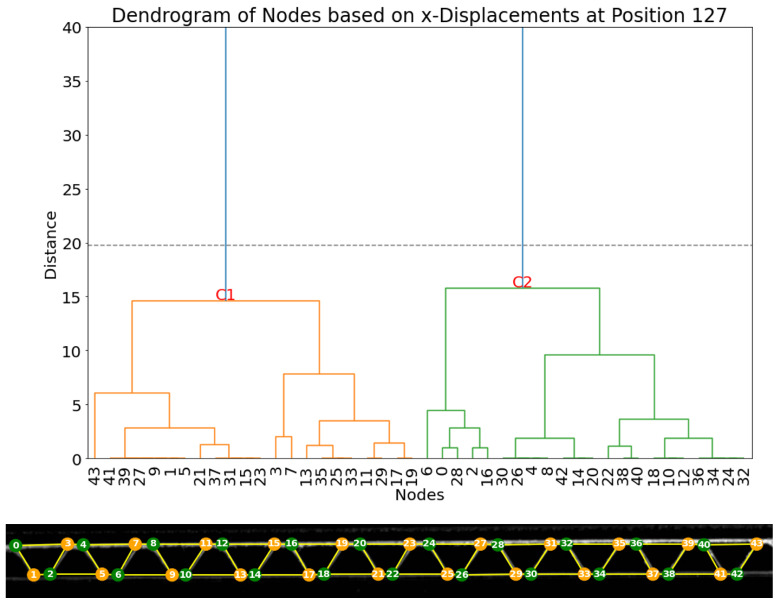
(**Top**): dendrogram and resulting clustering of the nodes based on horizontal displacement $d_{x}$ in frame 127. (**Bottom**): visualization of the clusters (cluster 1: orange; cluster 2: green).

A second clustering method is based on the analysis of the horizontal displacements, whose resulting dendrogram is represented in Figure 15. Here, two main clusters can be separated. Cluster C2 contains the nodes on the right side in each pair, and a second main cluster, C1, contains the nodes on the left side in each pair.

**Figure 16:**
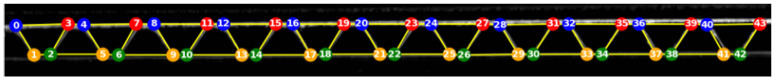
Visualization of the resulting four subgroups based on the combination of the pairs of clusters resulting from the vertical and horizontal displacements.

The two clustering methods, based on the horizontal and vertical displacements, provide two approaches for accurately forming two groups of nodes. The combination of these two methods, by creating sets of nodes based on the intersection of every two clusters, allowed for the creation of four distinct subgroups of nodes, as illustrated in Figure 16.

$$Subgroup_{1}=C1_{y}\cap C2_{x}\;Subgroup_{2}=C1_{y}\cap C1_{x}$$

$$Subgroup_{3}=C1_{y}\cap C2_{x}\;Subgroup_{4}=C2_{y}\cap C1_{x}$$

**Figure 17:**
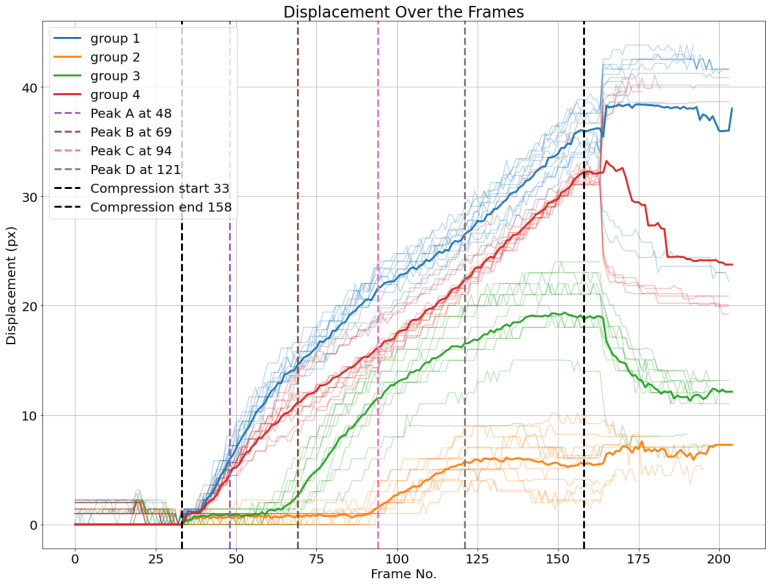
Euclidean displacements *d* of node groups. The average of each cluster per frame is plotted.

This way, all the nodes located periodically at the same position, labeled with top left (red), top right (blue), bottom left (orange), and bottom right (green), are separated into one subgroup. The formation of these four subgroups based on clustering the displacement curves shows that the nodes in all these subgroups experience similar displacements, which can be explained by the periodicity of the fluting medium. The nodes can be classified into two groups and four subgroups based on the different representations of displacements, i.e., absolute (or Euclidean), horizontal, and vertical displacements. Based on the last four subgroups, displacement averages are calculated for every subgroup, as illustrated in Figure 17. Additionally, the four load peaks are indicated in the corresponding frame position.

One can observe that the average displacement curves of groups 1 and 4, corresponding to the nodes over the upper liner, start developing from the beginning of the compression phase, while groups 2 and 3, corresponding to nodes over the lower liner, start developing later, nearly at the same time as the load peaks are measured. The localized buckling of fluting segments causes the flattening of the fluting tips in contact with the lower liner, which is characterized by the displacement of nodes on this liner. However, there are no clear features that characterize peaks A and D based on the displacement curves. The start of the movement can be detected by a displacement threshold of the average displacement curves of different groups set at 1.5 pixels.

Moreover, it is possible to observe from peak B that bottom-left nodes and bottom-right nodes have different Euclidean displacements. This means that the deformation does not happen symmetrically. While this is expected due to the geometric imperfections, this adds some relevant information that cannot be concluded based solely on the load–deformation curve.

**Figure 18:**
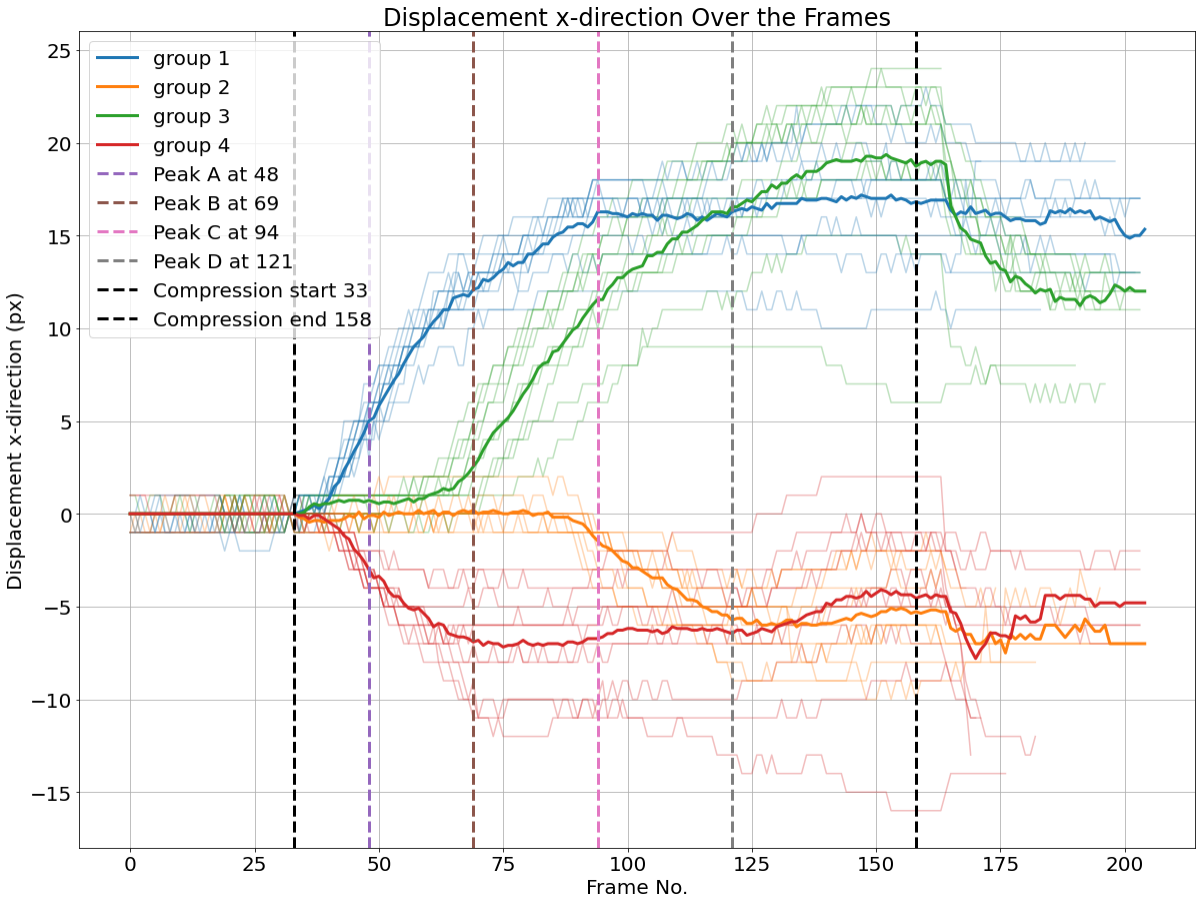
Horizontal displacements $d_{x}$ of node groups. The average of each cluster per frame is plotted.

The same can be applied based on the horizontal displacements of nodes. First, averages of horizontal displacements of different groups are calculated. Then, thresholds of 1.5 and −1.5 pixels are set to detect the start of movement as illustrated in Figure 18.

**Figure 19:**
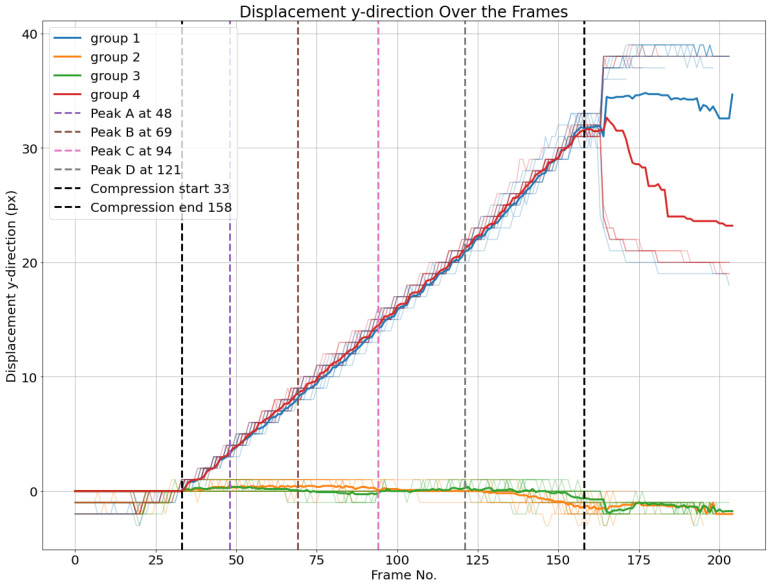
Vertical displacements $d_{y}$ of node groups. The average of each cluster per frame is plotted.

Additionally, the averaged vertical displacement for each of the four groups is illustrated in Figure 19. Groups 1 and 4, comprising nodes over the upper liner, have nearly identical vertical displacements in the compression phase. The same applies for groups 2 and 3 of nodes over the lower liner, which have nearly no vertical displacement.

**Figure 20:**
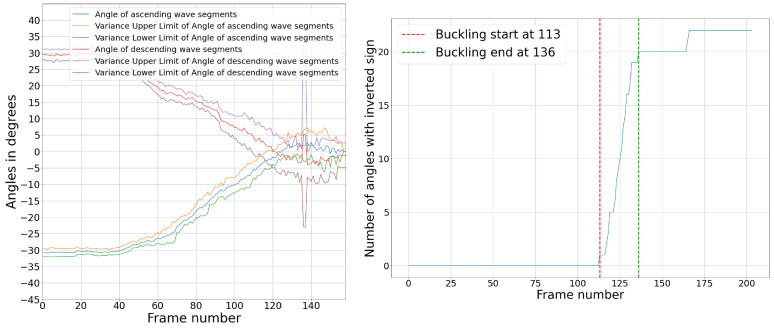
(**Left**): angle variation over the frames, per segment group. (**Right**): buckling analysis.

The highest load peak, D, was predicted in another approach based on the configuration of the board, which is characterized in this last peak by short vertical columns in the fluting medium. The load drop after this peak is caused by the buckling of the vertical fluting columns with an S-form. This causes the angles between the fluting column and the vertical axis in the thickness direction to switch signs. The average angle values are first calculated and illustrated in Figure 20. At the beginning, as the fluting is sine-waved, angles have values around 30° and −30° with the vertical direction. These values change toward zero during the compression as the fluting tips flatten and the middle fluting segments become more and more vertical. At a given point, they become totally vertical. This is where an angle equal to zero is measured. After this, the liners are very close to each other, and the fluting columns become too short, which causes errors in angle measurements. However, this is not relevant in this case, as the most important information to detect the peak position is the frames in which angles switch signs for the first time. The number of angles that switch signs in each frame is then calculated. Let us assume that when a fluting segment buckles, the angle with the vertical level switches signs. So, the buckling of the structure starts when the first segment switches its sign and ends when 90% of the total number of fluting segments switch signs. This range is illustrated in Figure 20.

**Figure 21:**
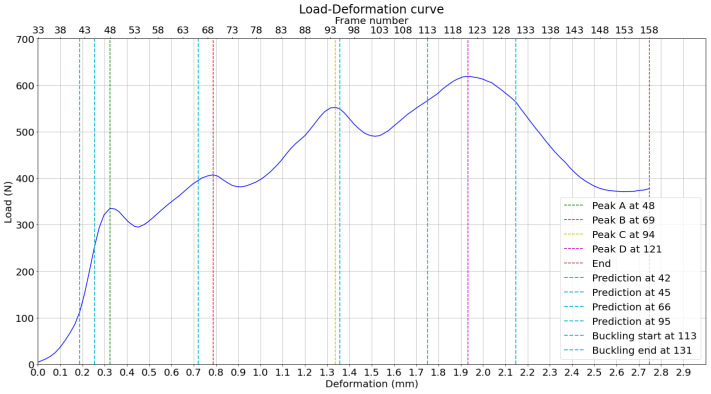
Load–deformation curve with measured and predicted load peaks, based on previous analysis.

The measured peak loads and the positions of their predictions based on the displacement curves in the horizontal direction are then plotted on the load–deformation curve of the compression test, as shown in Figure 21. It can be observed, through a direct observation of the bottom x-axis, that peaks A and B are predicted less than 100 μm of deformation before they happen. This attempt to predict the load peaks is based on averaged displacement curves, which reduce random noise and enhance the reliability of identifying consistent deformation patterns. The buckling range is around the highest peak, D, where the buckling is expected to happen.

### 4.2. Image and Graph Analysis: Experiment 2

**Figure 22:**
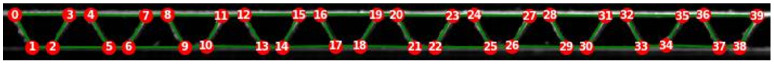
Graph visualization of the specimen of the second experiment.

**Figure 23:**
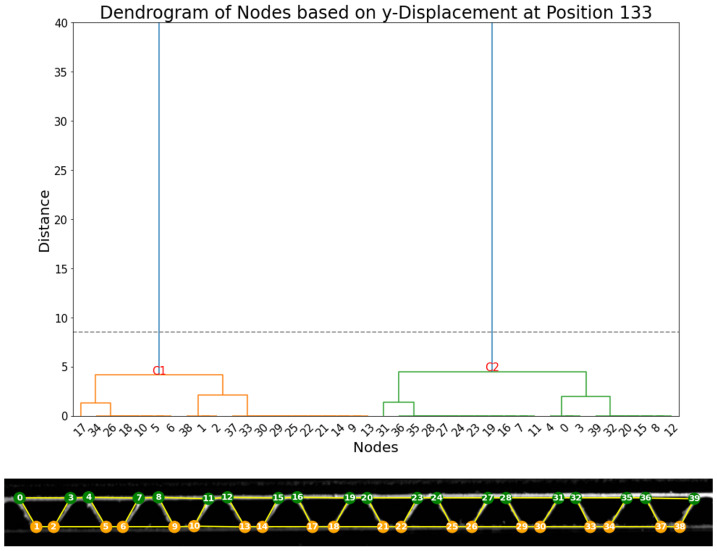
(**Top**): dendrogram and resulting clustering of the nodes based on vertical displacement $d_{y}$ in frame 133. (**Bottom**): visualization of the clusters (cluster 1: orange; cluster 2: green).

In this new specimen (Figure 22), 40 nodes were detected and tracked over the frames of the compression video. Firstly, the vertical displacements were analyzed. The dendrogram of displacements at frame 127, as shown in Figure 23, provides evidence for the existence of two principal clusters of nodes (C1 and C2). As observed in the first experiment, two main clusters align with the two expected groups of nodes perfectly, as cluster C1 contains all 20 nodes within the lower liner and all 20 nodes within the upper liner.

**Figure 24:**
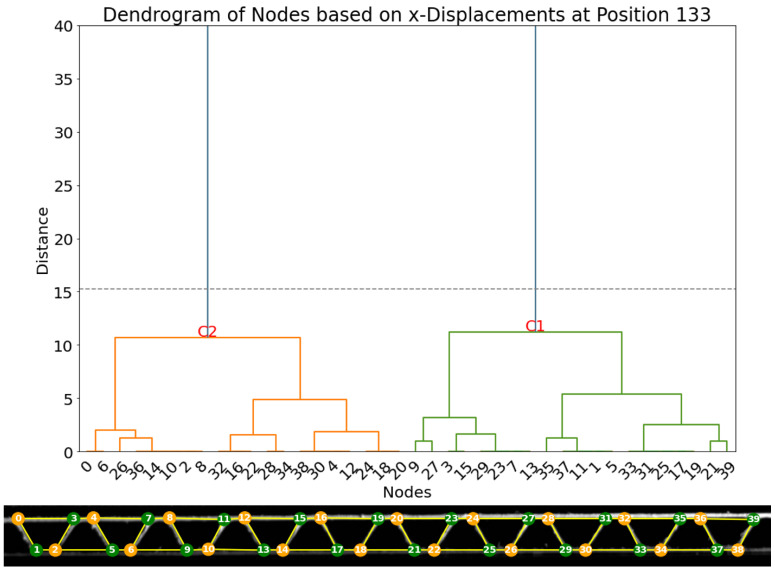
(**Top**): dendrogram and resulting clustering of the nodes based on horizontal displacement $d_{x}$ in frame 133. (**Bottom**): visualization of the clusters (cluster 1: green; cluster 2: orange).

A second clustering method is based on the analysis of the horizontal displacements, whose resulting dendrogram is represented in Figure 24. Here, two main clusters can be separated in the same way as experiment 1.

**Figure 25:**
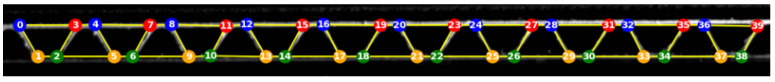
Visualization of the resulting four subgroups based on the combination of the pairs of subgroups resulting from the vertical and horizontal displacements.

The two clustering methods, based on the horizontal and vertical displacements, provide two approaches to accurately forming two groups of nodes. The combination of these two methods, by creating sets of nodes based on the intersection of every two clusters, allowed for the creation of four distinct subgroups of nodes, as illustrated in Figure 25.

**Figure 26:**
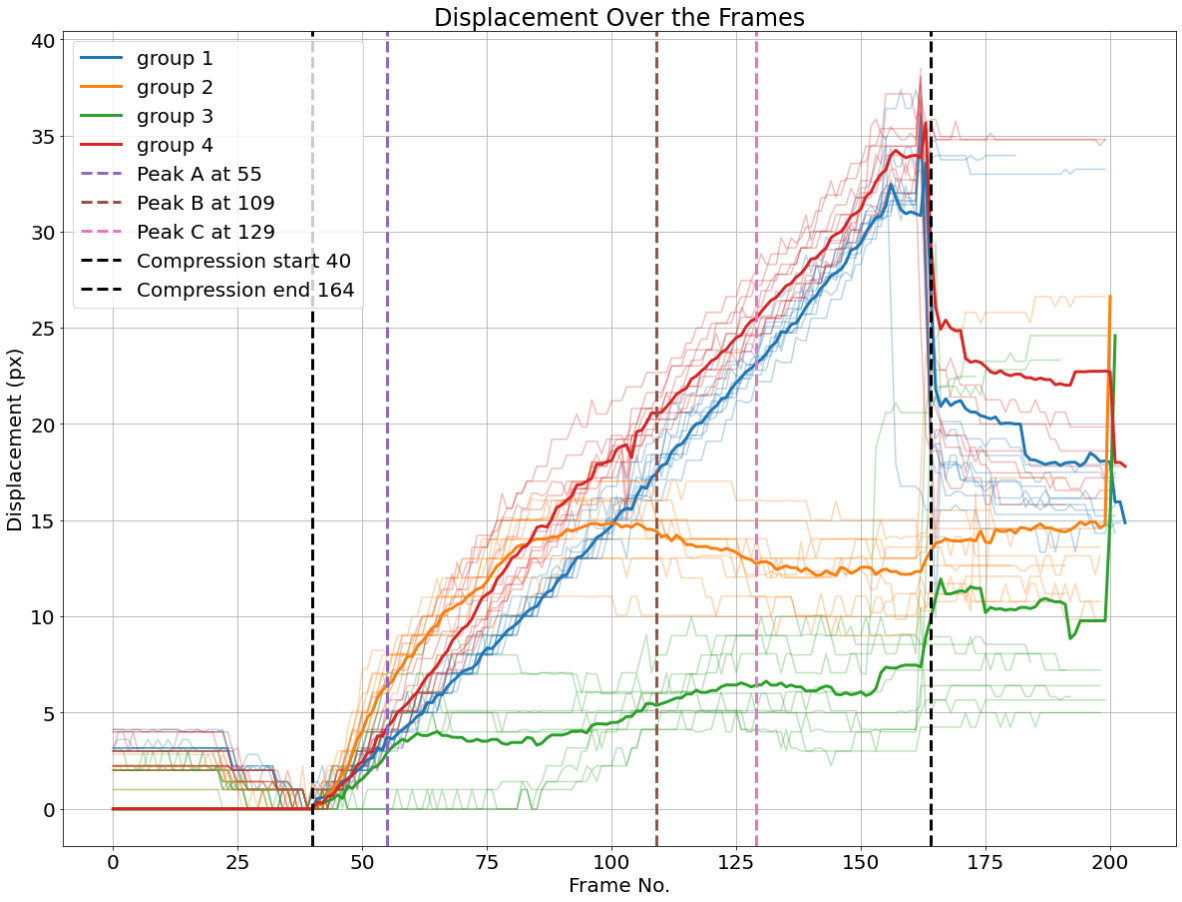
Euclidean displacements *d* of node groups. The average of each cluster per frame is plotted.

Based on the last four subgroups, displacement averages are calculated for every subgroup, as illustrated in Figure 26. Additionally, the four load peaks are indicated in the corresponding frame position.

One can observe that the average displacement curves of all groups start developing from the beginning of the compression phase, with groups 1 and 4, corresponding to the top liner, seeming not significantly different from each other in this perspective, and with the orange group (group 2) having a significant displacement, likely all of it corresponding to the horizontal displacement, as bottom liner nodes do not move vertically. This may suggest a significant move of the board to the left at the beginning of the compression, which is likely the way the structure becomes more stable for the rest of the compression.

**Figure 27:**
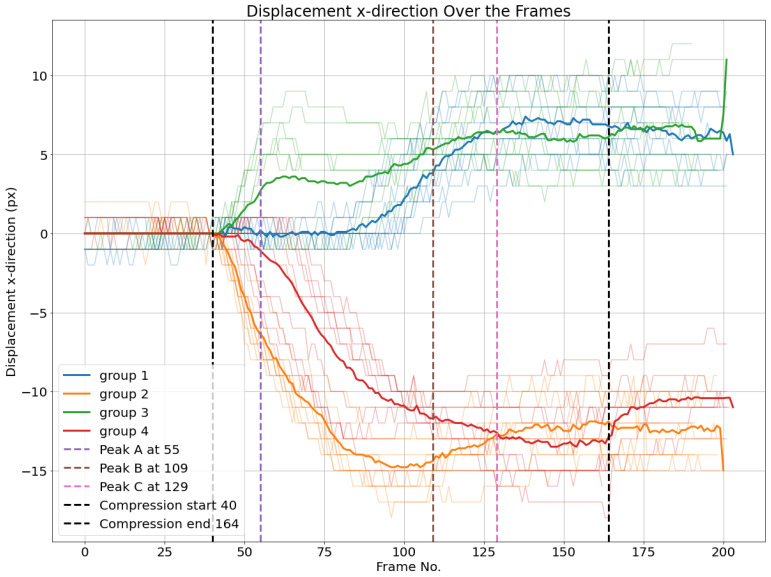
Horizontal displacements $d_{x}$ of node groups. The average of each cluster per frame is plotted.

The same process is applied based on the horizontal displacements of nodes. First, averages of horizontal displacements of different groups are calculated. Then, thresholds of 1.5 and −1.5 pixels are set to detect the start of movement, as illustrated in Figure 27.

While in experiment 1, it was possible to verify that upper liner nodes started to move horizontally first before the first peak compared to the nodes in the bottom liner, in experiment 2, it happened the opposite—in fact, bottom liner nodes were faster before the first peak, and then the top liner nodes. What is also interesting to observe is the fact that peak B happens after the last group to move horizontally, unlike in group 1. There is still another observation that is also possible to observe in Figure 27 in common with Figure 18 is that when the average of groups 1 and 3 intersect at the same time as groups 2 and 4, it also coincides with the last load–deformation curve peak. More experiments with different geometries may be necessary to explain it better.

**Figure 28:**
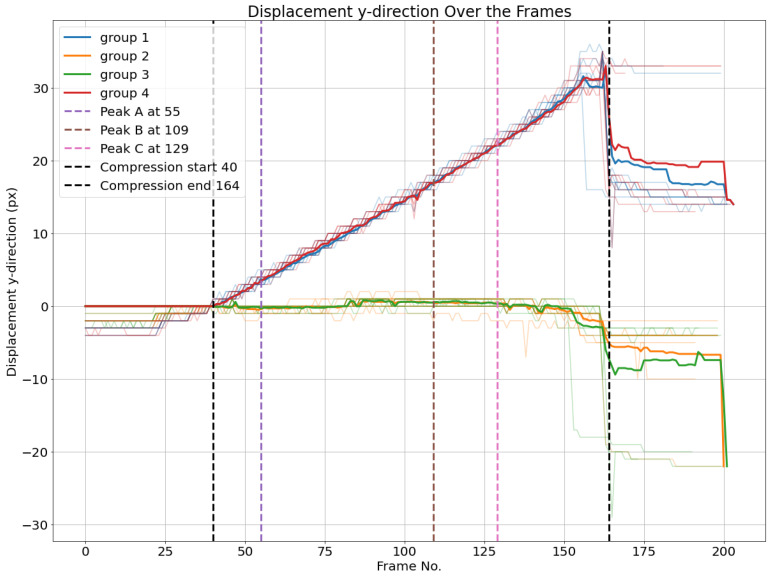
Vertical displacements $d_{y}$ of node groups. The average of each cluster per frame is plotted.

Additionally, the averaged vertical displacement for each of the four groups is illustrated in Figure 28. The behavior here identified is very similar to what had already been observed in experiment 1 (Figure 19).

**Figure 29:**
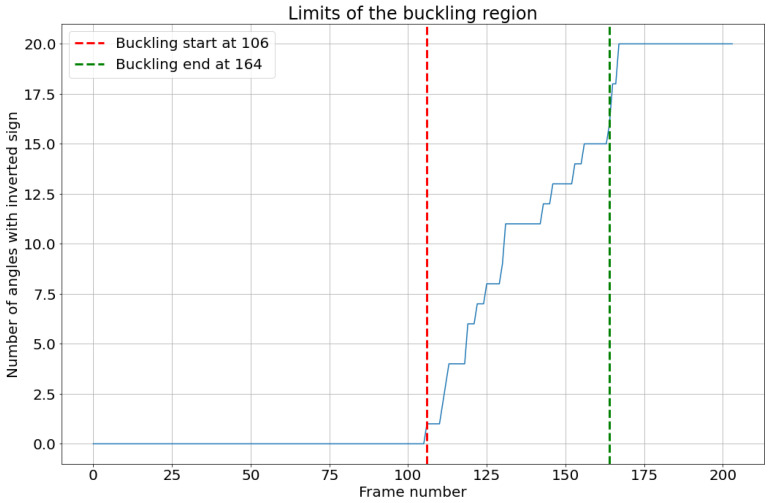
(**Left**): angle variation over the frames, per node group. (**Right**): buckling analysis.

As in experiment 1, the number of angles throughout the flute that switch signs in each frame was then calculated. Let us again assume that when a fluting segment buckles, the angle with the vertical level switches signs. So, the buckling of the structure starts when the first segment switches its sign and ends when 80% of the total number of fluting segments switch signs. This range is illustrated in Figure 29.

**Figure 30:**
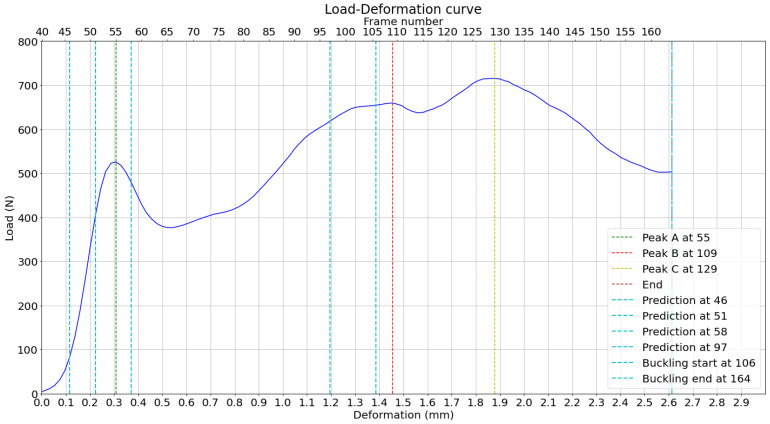
Load–deformation curve with measured and predicted load peaks, based on previous analysis.

The measured peak loads and the positions of their predictions based on the displacement curves in the horizontal direction are then plotted on the load–deformation curve of the compression test, as shown in Figure 30. It can be observed, through a direct observation of the bottom x-axis, that peaks A and B are predicted before deformation. This attempt to predict the load peaks is based on averaged displacement curves, increasing reliability. The buckling range is around the highest peak, C, which is much higher than in experiment 1.

### 4.3. Comparison of the Experiments

One of the goals of this research was to show that one can gain more significant information and prediction capability of the compression behavior of a corrugated board using graphs. Both experiments were undertaken based on two different specimens, but with exactly the same expected geometry. From the results, it is possible to observe that the impact of geometric imperfections is significant and not observable solely with the load–deformation curve.

Common observations between both experiments are as follows:

- The formation of the subgroups was maintained by means of the clustering analysis.
- Both experiments could cluster upper and bottom nodes using the vertical displacement, and the left-leaning and right-leaning node groups could be clustered the best using the horizontal displacement.
- The last peak happens in both situations when left-leaning and right-leaning node groups have the same displacement at the same time.
- The nodes do not move symmetrically.

However, differences between experiments were observable:

- Experiment 1 has three different trends visible in Euclidean displacement, while Experiment 2 has two different trends, which might justify the difference in the number of load peaks.
- Experiment 1 has more positive horizontal displacements (movement of the nodes to the right), while Experiment 2 has more negative horizontal displacements (movement of the nodes to the left).
- In Experiment 1, nodes move slower horizontally compared to in Experiment 2.

### 4.4. Answering the Research Questions

Graphs proved to be advantageous in reducing the dimensionality of corrugated board images while preserving the most important information, which is the profile geometry during a compression test. Every frame of the videos recorded during the compression tests was replaced with a graph with a limited number of nodes and edges. This provided a reasonable amount of data that can be analyzed more efficiently than raw images. Although each image was 44.3 ± 14.3 KB in size, each graph required only 4 ± 1 KB, achieving a tenfold reduction in data size (*p* < 0.05). This reduction was already expected, as most of the pixels represent background that is not relevant for the compression analysis, while the nodes and connectivity information that constitute the graph is the most relevant information and it is lighter information to store. Even when considering a larger set of nodes, the image will always contain more information, as the number of nodes cannot exceed the number of pixels.

Moreover, the results show that it is possible to collect multiple displacement curves from the nodes, and multiple clusters representing different compression behaviors could be analyzed. There is clearly a movement of the flute to one of the sides, which is not seen in the load–deformation curve only. In the first experiment, the nodes moved more to the right, while in the second experiment, they moved more to the left. This is important information that could be retrieved from the analysis carried out in the current study, as it can be useful for the prediction of the mechanical behavior, gaining more information than analyzing the load–deformation curve.

The following aspects were examined in both experiments:

- The average of two out of the four sub-clusters started to make a significant horizontal displacement before the first peak happened.
- The best reasonable predictors are observed using the horizontal displacement, but the vertical displacement is highly relevant for a different segmentation, allowing for the formation of the four sub-clusters.
- All nodes have a significant displacement, on average, before buckling happens.

### 4.5. Limitations

The proposed procedure depends, to a large extent, on the image quality of the corrugated profile. However, the filtering input must be manually adapted for different samples to obtain optimal results. This can be improved and made more automatic by developing an adapted filtering process to eliminate the delaminated paper fibers. Automated and more robust filtering can improve the algorithm results and make it applicable to more samples with different qualities and imperfections. The experimental setup can be optimized so that videos are recorded under uniform light conditions. The profile geometry can be approximated more accurately using more nodes and edges to describe the corrugated medium and better preserve the curvatures of the sine wave. Moreover, hierarchical clustering was considered for the analysis of the current experiments, which were conducted with a small number of nodes. Hierarchical clustering can be replaced with K-Means, HDBSCAN, or even approximated hierarchical clustering for a larger number of nodes, when such application justifies. Finally, the node tracking algorithm is based on policies that must be better explored in the future in order to make it more robust to potential node outliers that can be much more difficult to tackle than those observed in these experiments. This might include, for instance, setting minimum and maximum distances between nodes or even by starting with an expected node configuration. In this regard, there are several points to explore in the future.

## 5. Conclusions

A graph-based image analysis of corrugated boards under compression is proposed as a new approach in this field, which helps to understand the compressive behavior of corrugated boards based on their cross-section images. The profile geometry is approximated using graphs. Node tracking is implemented to associate nodes that represent the same regions in different frames with each other.

Based on a graph analysis consisting of the displacement computation of nodes, different groups of nodes were clustered. The average displacement of each group was then calculated and used to predict the load peaks in the load–deformation curve corresponding to the buckling of the structure. The prediction of three load peaks out of four, in the case of experiment 1, was relatively precise. Node displacements over time provided new insights into the corrugated board deformation analysis. It is possible to observe when and where deformations in the board are occurring and how buckling is spreading.

Improving knowledge about the mechanical behavior of corrugated boards using graph-based analysis and the modeling of images of the real product could provide more opportunities for shape and topology optimizations, and applying graphs to approximate the profile geometry reduced the dimensionality of image data. This analysis with graphs can be also extended to other structures and other materials in future applications.

The limitations can be addressed in future work by developing an improved filtering process and improving the precision of the geometry approximation by adapting the code and adding more nodes. This study represents the first application of graphs in the field of corrugated boards. Graphs can be useful in future research to apply machine learning techniques for the purpose of design optimization. This underscores the need for more experiments and a greater variety of geometries to be tested.

Moreover, replacing the graph with a beam structure model can allow for a rapid simulation of the board deformation under given load and boundary conditions. Regions where stresses are maximized could then be identified. This can also be tackled in future works.

## Figures and Tables

**Table 1:** Corrugated board sample dimensions.

Flute Type	H (mm)	λ (mm)	*t_f_* (μm)	*t_l_* (μm)
C	4.0 ± 0.1	8.0 ± 0.2	250 ± 80	250 ± 80

**Table 2:** Parameter settings for image processing filters.

Parameter Set	Gaussian Filter Kernel	Median Filter Kernel	Bin. Threshold
(a)	1 × 1	1 × 1	25
(b)	5 × 5	3× 3	50
(c)	5 × 5	3 × 3	25

## Data Availability

The original data presented in the study are openly available in GitHub at https://github.com/ricardofitas/Graphs_Analysis_Corrugated_Boards (accessed on 1 December 2024).
